# How conformational changes near the F pocket of MHC class I proteins mediate chaperone assisted peptide loading

**DOI:** 10.3389/fimmu.2025.1689803

**Published:** 2025-12-15

**Authors:** Simone Göppert, Sebastian Springer, Martin Zacharias

**Affiliations:** 1 Physics Department and Center of Protein Assemblies, Technical University of Munich, Garching, Germany; 2 School of Science, Constructor University, Bremen, Germany

**Keywords:** MHC class I, antigen presentation, peptide loading, tapasin, TAPBPR, peptide editing, chaperone assisted binding

## Abstract

**Background:**

Efficient recognition of antigenic peptides bound to major histocompatibility complex (MHC) class I molecules on the surface of cells by immune cells requires sufficiently stable peptide-MHC I complexes. Antigenic peptides of 8–10 amino acids are typically bound in a narrow cleft between two flanking α_1_ and α_2_ helices on top of an extended β sheet floor. For some MHC I alleles the efficient loading with high-affinity peptides in the endoplasmic reticulum (ER) requires the transient binding and assistance of the chaperone proteins tapasin and/or TAPBPR. The structures of both chaperones in complex with MHC I molecules have been resolved and indicate similar structural interface elements and also similar structural changes of the bound MHC I molecules which includes a significant shift of an α_2–1_ helix, a segment of the α_2_ helix, which partially opens up the binding cleft.

**Methods:**

The role of this α_2–1_ helix movement for the peptide loading and editing processes is not fully understood. We employed extensive Molecular Dynamics (MD) simulations and free energy calculations to estimate free energy changes associated with the α_2–1_ helix movement in the absence as well as presence of low- and high-affinity peptides and in complex with tapasin or TAPBPR.

**Results:**

The α_2–1_ helix shift with respect to the conformation in a native MHC I peptide complex significantly destabilizes the binding of peptides and can induce partial dissociation in case of low and medium-affinity peptides. Only a bound high-affinity peptide leads to a narrowing of the binding cleft and reduces the interaction of the MHC I with the chaperone molecules.

**Conclusions:**

The simulations indicate that the conformational shifts of the α_2–1_ helix with respect to the chaperone and the MHC I molecule play a dominant role for destabilizing peptide binding as well as triggering release from the chaperone in case of high-affinity peptide binding. In addition to the role of the α_2–1_ helix, we also compared the motion of a loop region found near the N-terminus of tapasin and TAPBPR that may also play a role in the chaperone process.

## Introduction

Major Histocompatibility complex class I (MHC I) proteins play a crucial role in the immune system by allowing it to monitor the internal proteome of cells (1, 2). MHC I molecules bind intracellular peptides in the Endoplasmic Reticulum (ER) and present them on the cell surface (1, 3–5). Peptides of a typical length of 8–10 amino acid residues are bound in a deep cleft of the heavy chain (HC) subunit between two (α_1_/α_2_) helices at the top of an extended β-sheet. The MHC complex also includes a smaller β_2_m subunit. To function effectively, MHC I proteins need to bind peptides with sufficiently high affinity because bound low-affinity peptides can be lost during transport from the ER to the cell surface (4, 6–9). On the cell surface, the stable MHC I - peptide complexes that are resistant to dissociation can be recognized by T cell receptors (TCR) on immune cells to stimulate T cell responses and can trigger apoptosis of infected or aberrant cells.

The genes encoding the HC are polymorphic (10), resulting in a variety of allotypes that only bind a subset of the potential antigenic intracellular peptides due to their individual and specific binding motif (1, 11, 12).

Peptide binding is mediated by conserved contacts between the side chains of the MHC I molecule and the backbone elements of the peptide as well as the occupation of two or more specificity pockets (e.g., the A, B and F pockets) located in the α_1_/α_2_ superdomain, into which the side chains of anchor residues sterically fit (13, 14).

Peptide selection in the ER is an iterative process that involves the class I-specific chaperone proteins tapasin (3, 15–17) and TAP binding protein-related (TAPBPR) (18–20). TAPBPR and tapasin are homologous and adopt similar three-dimensional (3D) structures. Whereas tapasin is associated with the TAP transporter and additional protein components of the peptide loading complex (PLC), TAPBPR acts independently in peptide loading and editing in the ER. Both chaperones form similar complexes with MHC I molecules and ensure that only high-affinity peptides are bound to MHC I molecules and are presented at the cell surface.

**Figure 1:**
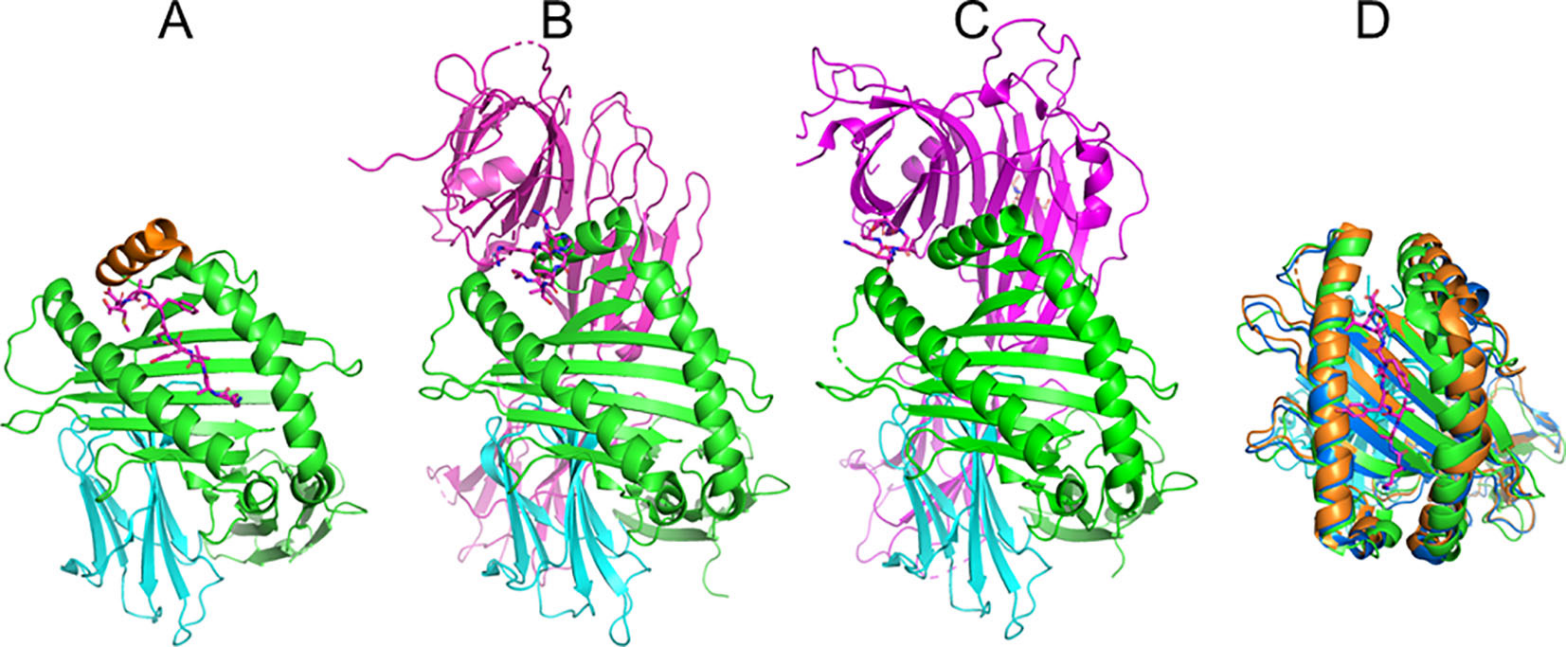
Structure of MHC class I proteins and complexes with TAPBPR and tapasin. **(A)** Illustration of MHC class I structure (H2-Db) with the heavy chain (green) and β2m subunit (light blue) as cartoon and a bound N-terminal peptide in stick model (mangenta). The α_2–1_ helix is colored orange. **(B)** MHC I (H2-Db) complex with TAPBPR (mangenta cartoon, pdb5opi). The scoop loop segment of TAPBPR is illustrated as stick model **(C)** MHC I (H2-Db) in complex with tapasin (mangenta cartoon, pdb7qng). Residues of the N-terminal loop region are shown as stick model. **(D)** Superposition of the MHC I peptide binding groove of native peptide bound MHC I (green, peptide in mangenta), in the complex with TAPBPR (blue) and tapasin (orange) to illustrate the movement of the α_2–1_ helix segment.

In recent years, the crystal structures of MHC I bound to TAPBPR have been solved (21, 22), and since 2022, also in complex with tapasin (6, 8). Both proteins share similar binding motifs and binding interfaces with MHC I molecules (illustrated in **Figure 1**). The interaction involves an extended concave surface of the chaperone that contacts the α_2–1_ helix, a segment of the α_2_ helix of the MHC I partner, on one side. The interaction shifts the α_2–1_ helix and adjacent elements to open the peptide binding cleft (**Figure 1**). Additional contacts involve the β_2_m subunit and the HC α_3_ domain. TAPBPR and tapasin contain a loop segment near the N terminus that in the complex with MHC I molecules is located above the F pocket binding region. The role of the N-terminal loop (also termed scoop loop or editing loop by some authors) is still not completely understood. Whereas the short loop is well-structured in the complex with tapasin (6, 8) it is represented by a weaker electron density in the complex with TAPBPR (21, 22). The loop of tapasin appears too short to allow direct contact with the F pocket, and a central hydrophobic Leu_18_ residue points towards the F pocket, but the side chain does not reach into the pocket in a fashion similar to a peptide with a F pocket residue of a bound peptide. In TAPBPR, the limited electron density indicates flexibility of the loop and a possibly transient, but not stable occupation of the F pocket by the loop segment.

Several biochemical and mutagenesis studies (23–25) and NMR studies (26, 27) indicate that the N-terminal loop could play a functional role in the control of peptide binding to class I molecules and in the editing process. For TAPBPR, substitution or deletion of the Leu_30_ residue in the loop region affects chaperone function (23). Substitution of Leu_18_ and Lys_16_ in tapasin affects the allele dependence of tapasin function (24). However, recent combined NMR, mutagenesis and MD simulation investigations indicate that the N-terminal loop of TAPBPR adopts conformations that hover above the MHC I groove, not reaching into the F pocket, instead promoting the capture of incoming peptides (28). In addition, the contribution of individual residues has been studied by deep mutational scanning methods, resulting in the identification of additional interface residues important for binding and peptide editing (26, 29).

As already indicated, interestingly, in both chaperone/MHC class I complexes, a considerable shift of the mobile α_2–1_ helix towards a more open geometry of the bound class I molecule is observed (**Figure 1**). It concerns especially the F pocket and C pocket regions. Indeed, previous studies suggested that the mobility of this α_2–1_ helix segment depends on the MHC allele (30–33) and is potentially an important element for chaperone function. Increased accessibility for peptide loading could be one function of a more open peptide binding cleft in the chaperone complexes. However, it is possible that the more open binding cleft in the presence of chaperones destabilizes peptide binding (to the C and F pocket), and that this leads to rapid dissociation of low affinity peptides. Hence, only peptides with sufficient binding affinity even to a deformed MHC binding cleft will have sufficient life time to prevent premature peptide dissociation before chaperone dissociation. Indeed, recently a TAPBPR variant with increased binding affinity to MHC I has been designed (34). This variant, termed TAPBPR^HiFi^, has full chaperone function even with a complete deletion of the N-terminal scoop loop region but still induces an outward shift of the α_2–1_ helix segment (34). However, the molecular mechanism of how the α_2–1_ helix movement affects or competes with peptide binding is still not completely understood.

In the present study, we focus specifically on the role of the mobile α_2–1_ helix and how it is influenced by peptide association in the F pocket region. We employ both unrestrained Molecular Dynamics (MD) simulations and advanced sampling techniques to investigate the mobility of the α_2–1_ helix in MHC I in native or chaperone-bound states and how it depends on the presence of low- or high-affinity peptides in the MHC I binding groove.

Our simulations indicate that the presence of a high-affinity peptide increases the tendency for movements of the α_2–1_ helix towards a more closed F pocket region but not reaching the narrow size found in peptide bound native MHC I molecules. This effect is not observed in case of a bound low-affinity peptide but, in contrast, the tendency for dissociation beginning at the C-terminus is significantly increased in chaperone bound complexes compared the case of a native MHC I protein. Our free energy simulations indicate further that the conformational shifts of the α_2–1_ helix segment alone can have a dramatic effect on high-affinity vs. low-affinity peptide binding. For the TAPBPR chaperone, we find that in the absence of a peptide, the central hydrophobic Leu_30_ of the N-terminal loop can reach into the MHC I-F pocket. However, it depends on the initial loop structure; starting from conformations outside the groove, no movement towards the floor of the MHC I-F pocket was observed during the MD simulations. Due to the smaller loop size in case of tapasin compared to TAPBPR, the central Leu_18_ residue in the N-terminal loop has a tendency to move towards the F pocket in case of no peptide or bound low-affinity peptide, but the Leu side chain cannot sterically reach the floor of the F pocket. The implications of our findings for understanding the mechanism of peptide exchange and editing will be discussed.

## Materials and methods

Experimental structures of mouse MHC class I allele H-2D^b^ in complex with TAPBPR (pdb-entry 5opi), in complex with tapasin (tpn) (pdb-entry 7qng), and in complex with a high-affinity peptide (pdb-entry 5swz) without chaperone served as start structures for Molecular Dynamics (MD) simulations. Missing loop segments in tpn and TAPBPR were built with AlphaFold2 (35). Start structures for chaperone-bound complexes including a bound peptide were generated by best superposition of the peptide bound reference structure onto the peptide binding cleft of the chaperone bound complexes. The coordinates of the peptide were added to the complexes and optimized using energy minimization (see below). *In silico* side chain substitution of the high affinity peptide was used to create start structures for complexes including low-affinity or intermediate-affinity peptides. The classification of the peptides into high-, intermediate-, and low-affinity was based on a combination of experimental evidence and rational design with an emphasis on modifying the residues that bind at or near the F pocket but preserving the peptide sequence that binds near the class I A pocket. This allows comparison of mobility or binding states near the F pocket during simulations without a direct modulation due to differences in the A pocket peptide sequence. The high-affinity peptide (ASNENMETM) is a well-characterized Influenza A virus epitope with extensive experimental validation as a strong H2-Db binder (see IEDB record, https://www.iedb.org/home_v3.php). The low-affinity variant (ASNENCKKG) was generated by substituting residues at the peptide C-terminus with amino acids predicted by NetMHCpan (36) to have minimal compatibility with the H-2D^b^ F-pocket, thereby reducing affinity while preserving the peptide backbone. The intermediate-affinity peptide (ASNENCKKA) differs from the low-affinity form by a single glycine-to-alanine substitution at the C-terminus, expected to moderately stabilize binding through improved van der Waals contacts at the F pocket.

However, we like to indicate that both Ala and Gly are disfavored at position 9 for the H-2D^b^ binding according to NetMHCpan. Hence, both modified peptides might be considered as low-affinity variants. Although there is no direct experimental proof for the affinity reduction of the generated peptides, we would like to emphasize that for other H-2D^b^ binding peptides, even the substitution of a single C-terminal residue (e.g. TAPDNLGY**M** vs. TAPDNLGY**A**, 37) to alanine strongly reduces binding. Hence, it is very likely that our low and medium affinity peptides with no or a small C-terminal side chain significantly reduce at least F pocket binding.

### Molecular modeling and dynamics simulations

The Amber20 molecular modeling software suite (38) was used for all energy minimization and MD simulations. Solvated starting structures were generated using the ff19SB (39) force field for proteins and the OPC water model (40, 41). The globular protein structures were embedded in octahedral water boxes with a minimum distance of 10.0 Å between the protein and the box boundary. Sodium and chloride ions were added to neutralize the charge of the systems and to obtain a ~100mM salt concentration followed by energy minimization (2500 steps). During all equilibration and production steps, long-range electrostatic interactions and vdW interactions beyond a 9 Å real space cut-off were calculated using the particle mesh Ewald method. Temperature control was maintained using the Langevin thermostat, with a collision frequency of 4.0 ps⁻¹. Pressure control was applied using isotropic scaling with the Berendsen barostat and a reference pressure of 1 bar. Additionally, the SHAKE algorithm was employed to constrain all bonds involving hydrogen atoms. The SETTLE algorithm (42) was used to constrain bond length in water molecules. The systems were heated in steps of 100 K to the final target temperature of 310 K within 0.5 ns and addition of positional restraints with a force constant of 12.0 kcal·mol^-1^·Å^-2^ with respect to the energy-minimized start structure. Positional restraints were applied to non-hydrogen protein atoms only. In a second phase, the force constants for positional restraints were gradually removed to 10 kcal/(mol·Å²) followed by 6 kcal/(mol·Å²), 3 kcal/(mol·Å²) and finally 0.25 kcal/(mol·Å²) within 1 ns. In the last stage, only the Cα atoms were positionally restrained. During heating and equilibration phases, a time step of 2 fs was used. To enable a time step of 4 fs for production runs, the hydrogen mass-repartitioning option (43) was employed. Production MD simulations were typically conducted using five replicas under NPT ensemble conditions with isotropic pressure scaling. The temperature was maintained at 310 K using Langevin dynamics with a time constant of 5 ps and a collision frequency of 4 ps⁻¹. The random seed for the Langevin thermostat was generated automatically (ig=-1). Each production replica was run with a time step of 4 fs using the Amber software package. For each replica, the simulation was run for a total of 1,000 ns of simulation time per replica. Pressure was controlled using the Berendsen barostat at a reference pressure of 1.0 bar with a relaxation time of 1.0 ps. In the case of maintaining an ‘unlocked scenario’ in an isolated MHC I molecule that is the arrangement of the α_1_ helix and the α_2–_1 helices kept close to the arrangement in the chaperone MHC I complex structure, we applied a weak distance restraint between the α_1_ helix and the α_2–1_ helix segments. The centers of mass of each segment were calculated using the Cα atoms of residues 75-86 (α_1_ helix segment) and residues 137-151 (α_2–_^1^ helix). A harmonic restraint with a force constant of 6 kcal/(mol·Å²) was applied to maintain a target distance of 16.0 Å between the two centers of mass representing the distance in the chaperone complex. All other simulation parameters were identical to those used in the standard MD simulations.

### Replica exchange umbrella sampling simulations

After completing the heat-up process, the equilibrated structures were used as the starting structures for a series of molecular dynamics simulations to prepare the system for replica exchange umbrella sampling (REUS). Initially, an unrestrained molecular dynamics run was performed to further relax the system. This simulation was conducted at 300 K using Langevin dynamics with a small collision frequency of 0.5 ps⁻¹ to maintain stable temperature control. The simulation was performed under constant volume conditions and ran for 25,000 steps with a 4 fs time step. To define the reaction coordinate for Umbrella Sampling, the center of mass for each helix segment was calculated using the Cα atoms from residue 75 to 86 for the α_1_ helix segment and the Cα atoms of residues 137 to 151 for defining the α_2–1_ helix. The center-of-mass distance was controlled with a force constant of 12.0 kcal/(mol·Å²) to gradually change the distance in a range of 12 Å to 18.5 Å in steps of 0.5 Å. This restrained simulation used the same conditions as the unrestrained run but extended to 250,000 steps to allow the system to relax at each specified helix distance. The resulting equilibrated structures from the pulling simulations were used as starting points for the subsequent REUS simulations. The final analysis phase involved repeating the restrained simulation three times, each consisting of 25,000 exchange attempts every 250 MD steps (1 ps) resulting in a total simulation time of 25 ns for each repeat. Acceptance rate for replica exchanges was > 20% for all REUS simulations.

### COM-distance determination and MMGBSA analysis

The distance between the α_1_ segment and α_2–1_ helix of the MHC was measured using the AmberTools suite (Amber20), specifically cpptraj. The distance calculation focused on the center of mass between two sets of residues: residues 137 to 151 and residues 75 to 86. Only the C-alpha (Cα) atoms of the residues within these regions were considered for the center of mass calculation. To ensure computational efficiency and manageability of the data, the measurements were taken every 10th frame from the trajectory.

The Molecular Mechanics Generalized Born Surface Area (MMGBSA) method was employed to estimate the binding free energy for each window of the Replica Exchange Umbrella Sampling (REUS) simulation using Amber20. To optimize computational efficiency while maintaining accuracy, every 20th frame for each window of the REUS simulation was used. In the MMGBSA analysis, only specific regions of the proteins were considered. For the MHC protein, only the α_1_ and α_2_ domains (residues 1-175) were included. For tapasin, the loop region (residues 14 to 24) was excluded, and for TAPBPR, the loop region (residues 21 to 35) was also excluded. The water and ions were removed. A modified Generalized Born model developed by Onufriev et al. (44) was selected (igb=2 in Amber input), with a salt concentration of 0.150 M, with the internal dielectric constant set to 1.0 and the external dielectric constant to 78.0, to reflect physiological conditions. The calculations focused on estimating the enthalpic interaction contribution to the binding free energy, and therefore, the conformational entropy contribution typically based on normal mode analysis was omitted.

## Results

### Unrestrained molecular dynamics simulations

**Figure 2:**
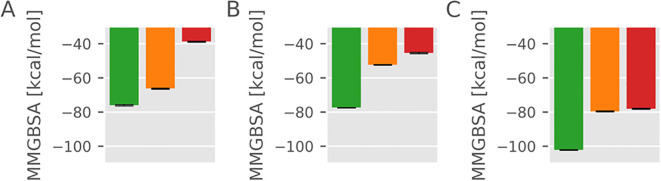
Calculated mean interaction energy using the MMGBSA approach between bound peptides. (green: high-affinity peptide, orange: intermediate-affinity peptide, red: low-affinity peptide) and MHC molecule: **(A)** MHC-peptide complexes bound to tapasin, **(B)** MHC-peptide complexes bound to TAPBPR, **(C)** native MHC-peptide complexes without bound chaperone. Error bars represents the standard error of the mean.

The structure and dynamics of the class I peptide binding region and especially the mobility of the α_2–1_ helix are influenced by bound peptides and by the association with the tapasin and TAPBPR chaperones. We performed multiple unrestrained MD simulations on a MHC class I H-2D^b^ molecule with either bound high affinity, medium affinity or low affinity peptides or simulations without a peptide. In addition, the same peptide-bound (or -unbound) states were generated for the class I molecule bound to either tapasin or TAPBPR (see Methods). For each case, 5 x 1000 ns simulations were performed. The high-affinity peptide corresponds to a sequence derived from the Influenza A virus nucleoprotein (sequence: ASNENMETM). The peptide that we term in the following low-affinity (ASNENCKKG) contains four mutations in the residues near the peptide C-terminus with residues known to exhibit low binding affinity for those specific positions according to predictions using NetMHCpan (36) to have minimal compatibility with the H-2D^b^ F pocket. It has the same 5 residue sequence at the N-terminus as the high affinity peptide to allow direct comparison of binding effects during simulations around the F pocket and to minimize any possible influence due to differences of the peptide region near the A-pocket region of the MHC class I molecule. The medium-affinity peptide (ASNENCKKA) is similar to the low-affinity peptide, except for the substitution of a glycine residue with alanine, which is expected to slightly improve its binding to the F pocket. The high-, medium-, and low-affinity peptides were selected or designed as described in the Methods, with substitutions at the C-terminus modulating F pocket interactions. In the starting structures, the peptides were all placed in a sterically optimal bound state in the MHC class I binding cleft. In all simulations, the MHC class I molecule and the complexes with either tapasin or TAPBPR remained stably bound with no tendency of chaperone dissociation or protein unfolding (**Supplementary Figure S1**). We also calculated the mean interaction between the bound peptides and the MHC molecule using the Molecular Mechanics Generalized Born Surface Area (MMGBSA) method (**Figure 2**). The estimated interaction free energies reproduce qualitatively the expected binding order for the low-, medium- and high-affinity peptides. Notably, the calculated affinities of the peptides to the native MHC are generally decreased when either TAPBPR or tapasin are bound, reflecting the impact of chaperone binding on peptide-MHC interactions. Hence, the calculations indicate that the chaperone induced MHC binding pocket deformation leads to a general decrease of peptide binding to the MHC binding cleft. It agrees qualitatively with experiments that indicate a significant reduction of peptide affinity to the MHC I groove in the presence of TAPBPR (~100 fold) (27). Despite the stability of the MHC molecule and bound chaperone during the simulations, peptide binding was not stable in every case, and partial dissociation of the low-affinity as well as medium affinity peptide was observed in extended simulations (see next paragraphs).

### Analysis of the F pocket size and α_2–1_ helix mobility

**Figure 3:**
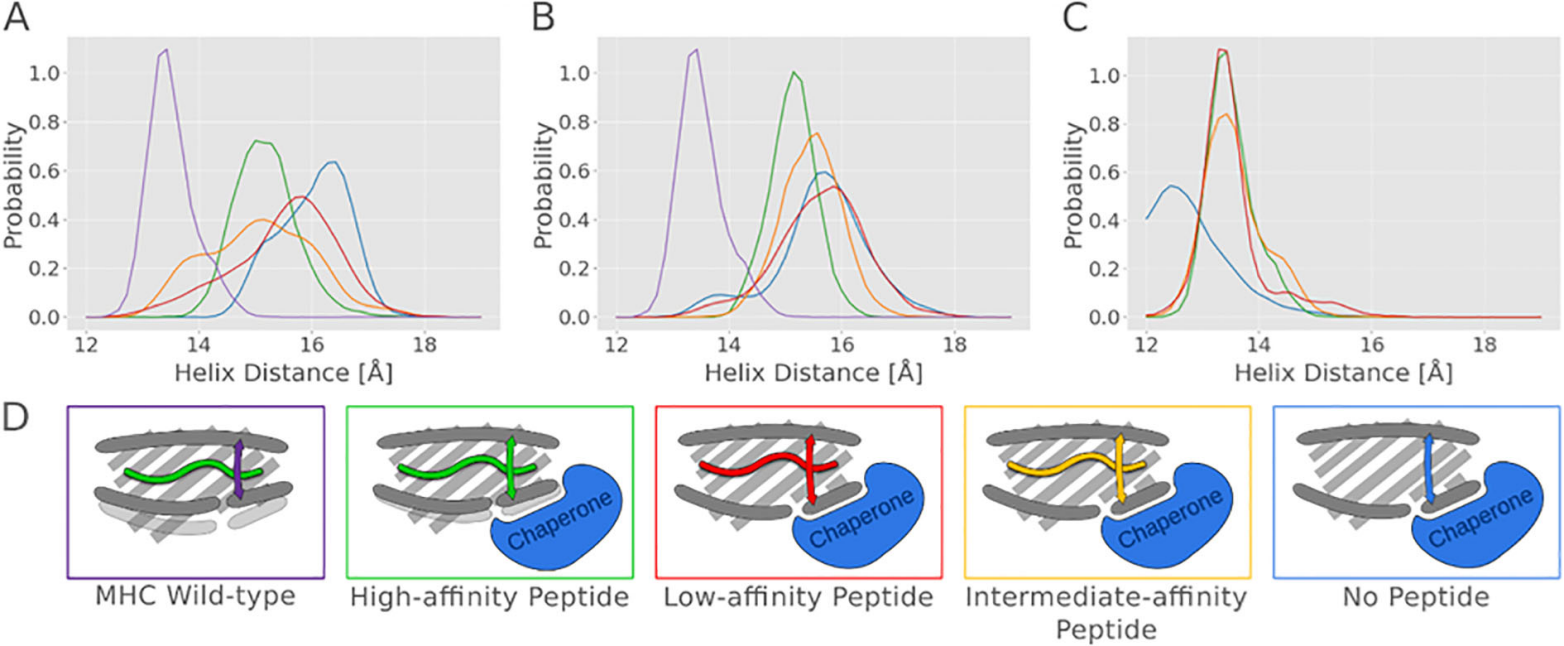
MHC helix dynamics in the presence of tapasin, TAPBPR and MHC in native conformation. The distance between the α_2–1_ and α_1_ helices was measured during free simulations and shown as histograms for different conditions: **(A)** Tapasin-bound MHC, **(B)** TAPBPR-bound MHC, and **(C)** native MHC I without chaperone. **(D)** Schematic illustration of the analyzed systems showing the position of the α_2–1_ helix and the bound peptide. The colors correspond to the different peptides: purple for MHC in native conformation, green for high-affinity peptide, red for low-affinity peptide, orange for intermediate-affinity peptide, and blue for the no-peptide, as the colors of the graph lines.

**Figure 4:**
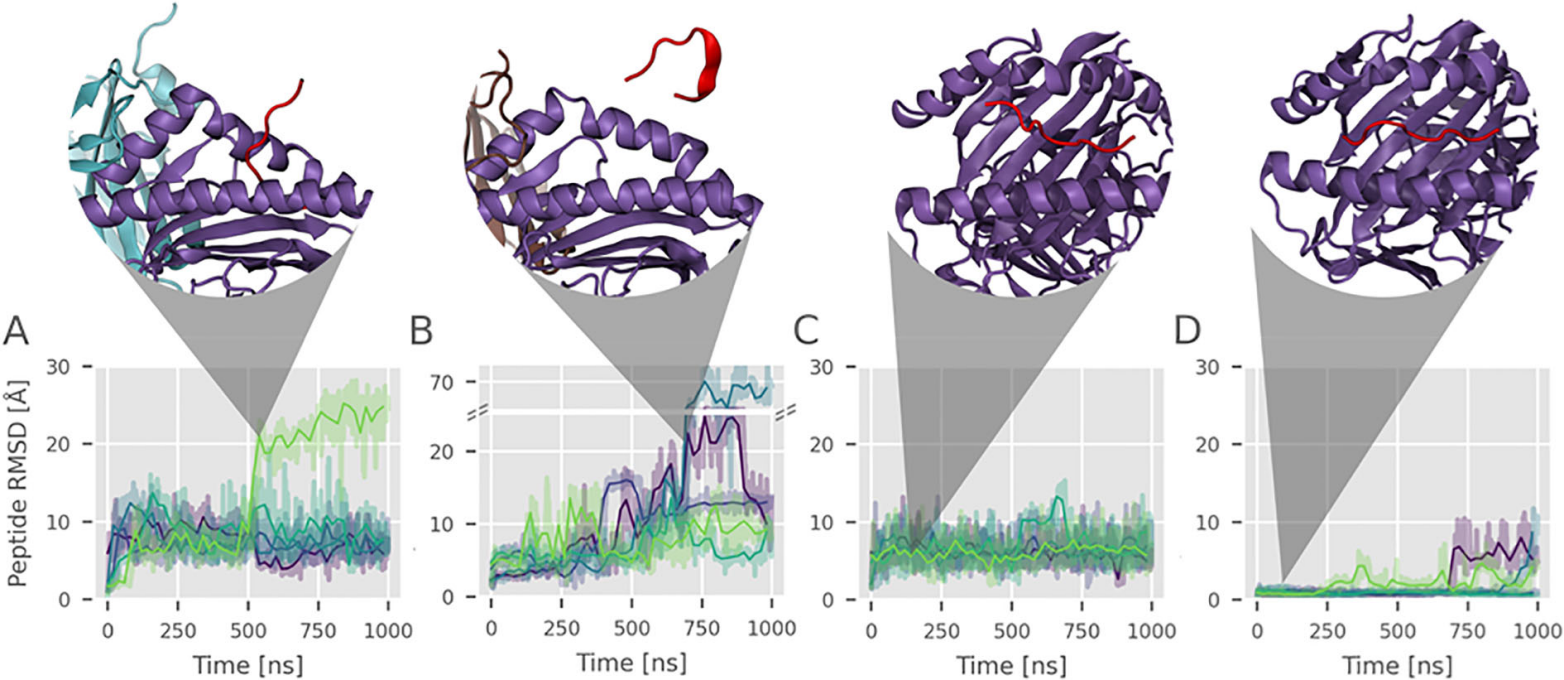
Low affinity peptide C-terminus shows increased flexibility upon chaperone binding and in the unlocked MHC state. Root Mean Square Deviation (RMSD) of the low-affinity peptide C-terminus (after best superposition of the HC binding cleft with respect to the start structure) vs. simulation time for five replicas in different conditions: **(A)** tapasin-bound MHC I, **(B)** TAPBPR-bound MHC I, **(C)** unlocked MHC I without chaperone, including weak restraints to keep the F pocket region in an open conformation similar to the structure in complex with chaperones. **(D)** Locked MHC I without chaperone, in native conformation with closed MHC I helices. Each colored line corresponds to one replica (replicas 1–5), with transparent lines representing raw data and solid lines showing the smoothed average (mean of 25 data points). The structural snapshots above the plots depict representative or interesting snapshots from the simulations.

The size of the binding cleft at the C and F pockets is determined by the distance of the flanking α_2–1_ helix relative to the α_1_ helical segment on the opposite side of the F pocket. In the case of the MD simulations of the native class I structure (without chaperones), we observed for the complexes with different peptides similar distance distributions with a mean (center-of-mass) distance between the helical segments of 13.5 ± 0.4 Å; for the case without bound peptide, the mean distance was smaller (12.6 ± 0.8 Å) (**Figure 3**). Only in simulations of the low- and intermediate-affinity peptides, a partial and transient dissociation near the F pocket was observed (see next paragraph and **Figure 4**, **Supplementary Figure S2**). In all cases with a bound high-affinity peptide, it remained stably bound in all simulations.

In contrast, in the simulations of the MHC molecule in complex with tapasin or TAPBPR, significantly larger distances between the α_2–1_ helix relative to the α_1_ helical segment were sampled. For the case without peptide, a mean distance of 16.0 ± 0.6 Å with bound tapasin or TAPBPR was sampled. In the case of a bound high-affinity peptide, the distance distribution was shifted towards a closer F pocket, but not reaching a helical segment distance as small as seen in the native MHC class I peptide complexes (mean distance 15.2 ± 0.5 Å, **Figure 3**). In case of the low affinity peptide, the distance distribution is similar to the case without bound peptide, and for a medium affinity peptide, an intermediate behavior is observed. The results indicate that the high-affinity peptide has the tendency to pull the α_2–1_ helix inward, although not reaching the α_2–1_ helix placement sampled in simulations with the MHC I in the absence of chaperones and a bound high-affinity peptide.

Interestingly, for the bound low-affinity peptides, a strong tendency for peptide dissociation (at the C-terminus) was observed in the tapasin and TAPBPR chaperone complexes, in contrast to a lesser tendency for the same peptide bound simulations in complex with MHC class I alone (**Figure 4**). A similar trend was seen for the intermediate-affinity peptide case (**Supplementary Figure S2**).

### The α_2–1_ helix displacement is sufficient to destabilize low-affinity peptide binding

To study the tendency of C-terminal peptide dissociation of the low-affinity peptide from the F pocket, we monitored the root mean square deviation (RMSD) of the last three peptide residues from the reference start placement (**Figure 4**). For all simulation replicas in the presence of bound tapasin or TAPBPR, we observe partial dissociation of the peptide C-terminus (**Figures 4a, b**).

In order to check that the increased tendency of peptide dissociation is indeed due to the increased α_2–1_ helix distance relative to the α_1_ helical segment, we next performed MD simulations starting from the structure as found in the complex with TAPBPR, but removed the TAPBPR and weakly restrained the distance between the helical segments to keep it close to the mean conformation found in the complex with the chaperone. This arrangement, in the following termed the ‘unlocked scenario’, completely excludes possible other contributions due to a chaperone bound to the MHC I molecule. Also, in this case, a strongly enhanced tendency for peptide dissociation at the F pocket was observed for 4 out of 5 independent simulations (**Figure 4c**). In contrast, for the native MHC I with bound low affinity peptide, the tendency of partial dissociation is reduced and occurs in part only in the final stages of 2 replica simulations (**Figure 4d**). This indicates that the opening of the F pocket region alone, as promoted by the chaperones, is sufficient to destabilize the binding of low-affinity peptides. A qualitatively similar tendency is observed in case of a bound intermediate-affinity peptide (**Supplementary Figure S2**). In contrast, for a high affinity peptide, the peptide C-terminus remains stably bound in all simulations (**Supplementary Figure S2**). The MHC α_1_ and α_2_ domains remained stable, during the simulations (RMSD vs. time, **Supplementary Figure S3**).

### Replica exchange umbrella sampling simulations

**Figure 5:**
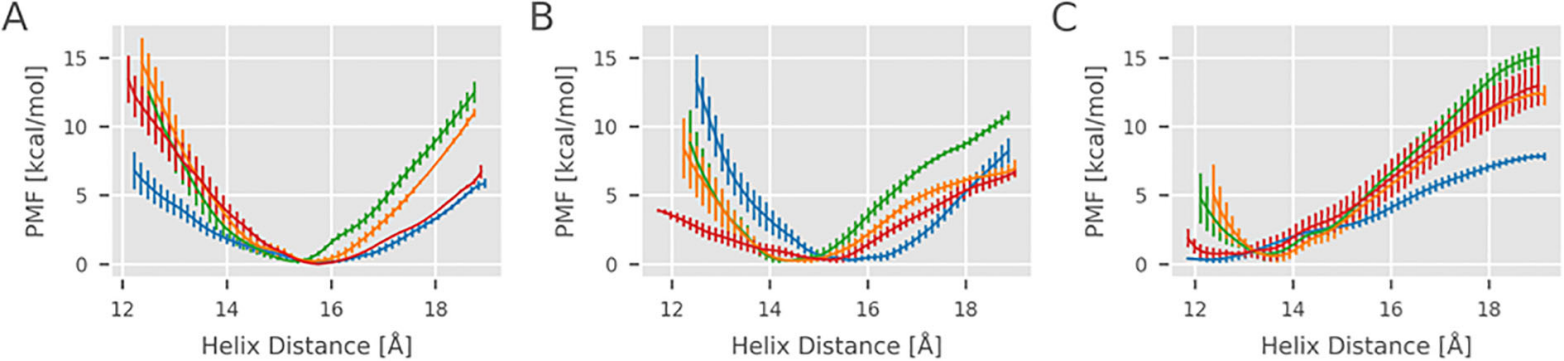
Free energy profiles of the helix opening from replica exchange simulations. The helix distance was increased from 12 Å to 19 Å using replica exchange simulations. The potential of mean force (PMF) is shown as a function of helix distance for **(A)** tapasin-bound MHC, **(B)** TAPBPR-bound MHC I and **(C)** native MHC I without chaperone. Each line color represents a different peptide: green for high-affinity peptide, orange for intermediate-affinity peptide, red for low-affinity peptide, and blue for the no peptide. Error bars indicate the standard error in each umbrella window.

The unrestrained continuous (c)MD simulations as described in the previous paragraph allowed us to obtain qualitative insights into the mobility of the α_2–1_ helix in the different peptide- and chaperone- bound states. In addition to multiple cMD simulations, we performed replica exchange umbrella sampling (REUS) simulations employing the center-of-mass distance between the α_2–1_ helix and the α_1_ helix segment flanking the F pocket as reaction coordinate (distance ranging from 12 to 18.5 Å, **Figure 5**). The REUS calculations allow us to estimate the free energy change associated with the helix displacement. In case of the native MHC class I complexes, the free energy change along the reaction coordinate indicated a free energy minimum close to the distance with highest probability found in the cMD simulations (**Figure 3c**). For a bound low-affinity peptide and for the empty class I cases, the minimum is shifted to smaller distances, and for the empty case, the increase of free energy along the reaction coordinate is also smaller compared to cases with bound medium- or high-affinity peptides (**Figure 5**).

In the complexes with tapasin and TAPBPR, the minimum free energy position is generally shifted to larger distances, as expected from the results of the multiple unrestrained cMD simulations. The free energy minimum obtained for a bound low-affinity peptide (15.2 Å) or in the absence of a peptide (15.8 Å) is shifted to larger helix distances compared to the case of medium- or high-affinity peptides. For the high-affinity peptide, the minimum free energy distance is shifted to 14.3 Å, and for the medium-affinity peptide to 14.4 Å. Additionally, the calculated free energy curve is less steep in the absence of a peptide or when a low-affinity peptide is bound. It is important to note that the free energy minimum distance even in case of a bound high affinity peptide is still larger than in the native MHC class I complex. The detected distance reduction with a high-affinity peptide compared to a low-affinity peptide might destabilize the interaction with the chaperone and might eventually induce dissociation of the MHC class I complex with a high-affinity peptide from the chaperone.

From the calculated free energy curves for the bound high-affinity peptide, it is possible to estimate the change of free energy to move the α_2–1_ helix from a 14.3 Å free energy minimum distance to the 15.8 Å equilibrium distance characteristic for the chaperone bound state without a bound peptide (which can be assumed to correspond to an optimum for the interaction with the chaperone). This estimate amounts to ~2.1-2.6 kcal/mol (free energy level of green curve at RC distance of 16 Å), which translates to a reduction of a binding constant of factor ~50.

### Interaction of tapasin/TAPBPR with heavy chain decreases when α_2–1_ helix is pulled inwards

**Figure 6:**
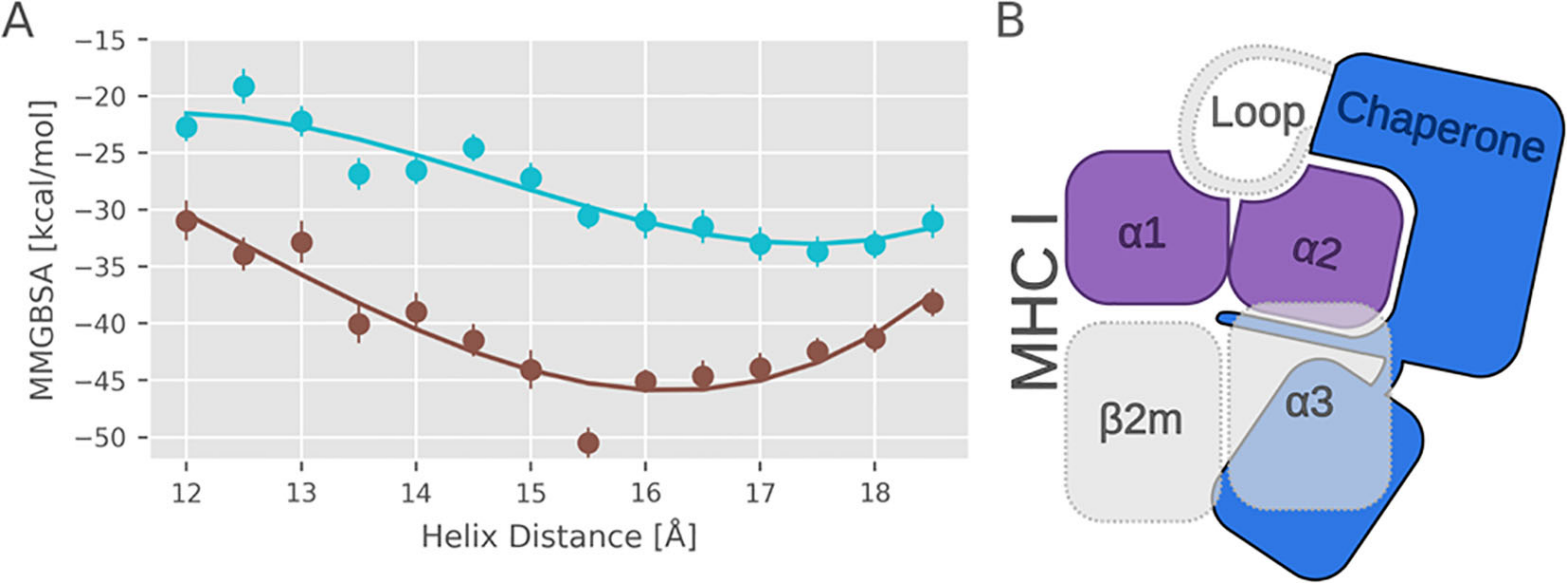
Binding affinity of the MHC I binding domain to tapasin and TAPBPR as a function of helix distance. **(A)** The graph shows the mean Molecular Mechanics Generalized Born Surface Area (MMGBSA) interaction energy between the MHC I binding interface and the chaperones tapasin (cyan) and TAPBPR (brown) for each replica exchange window. Calculations were performed in the absence of bound peptide to isolate the specific influence of α_2–1_ helix motion on chaperone interactions. Error bars are standard errors of the mean. **(B)** The scheme illustrates the structural setup used for the MMGBSA calculations. On the MHC I side only the HC α_1_-α_2_ peptide binding domain was included in the analysis, while on the chaperone side the loop regions were excluded to minimize structural noise and focus on the α_2–1_ helix binding interface.

We next asked whether the displacement of the α_2–1_ helix from the equilibrium position directly affects the interaction of the MHC I protein with the chaperones. We employed the MMGBSA approach to calculate the mean interaction energy between the MHC I binding interface and the tapasin or TAPBPR chaperone for each umbrella simulation window. In this approach, the solvent is taken into account implicitly, and it allows us only to estimate the change in interaction energy upon moving the α_2–1_ helix away from the equilibrium distance. The calculations were performed for the cases without bound peptide to assess the pure influence of the α_2–1_ helix movement (**Figure 6**), not counting possible changes in interaction between peptide and chaperone. Indeed, the results indicate that the interaction energy between the MHC I protein and the chaperones becomes less favorable when the α_2–1_ helix is pulled closer to the α_1_ helix segment, narrowing the F pocket. However, it also decreases when the helix is pulled excessively towards larger distances (enhanced F pocket opening) because of increased sterical overlap with the bound chaperone. The narrowing of the F pocket over the range sampled in the US simulations is accompanied by only a small average increase of ~1 Å in the distance between the α_2–1_ helix and the corresponding binding region on tapasin or TAPBPR (see **Supplementary Figure S5**). It indicates that the interaction between the MHC I protein and the chaperones is indeed perturbed by the binding of a high-affinity peptide, but a disruption likely followed by a dissociation of the class I molecule is not observed on time scale of the simulations. Consequently, binding of a high-affinity peptide in the MHC class I binding cleft induces a movement of the α_2–1_ helix towards a narrower F pocket. This in turn reduces the favorable interaction of the MHC I molecule with tapasin or TAPBPR chaperones, and the likelihood for dissociation is increased. However, on the time scale of our simulations, this latter process cannot be observed directly.

### Tapasin/TAPBPR N-terminal loop movement

Although the role of the α_2–1_ helix movement in chaperone-bound MHC I is at the focus of our study, we also looked at the role of a loop structure (called the scoop loop by some investigators) near the N-terminus of TAPBPR (residues 21-35), and a similar but smaller loop (residues 14-24) in tapasin located near and above the F pocket (**Figure 1**). To analyze the influence on peptide binding and stability, we recorded the RMSD of the loop backbone and analyzed the distance between the central loop Leu residue (Leu_18_ in case of tapasin, Leu_30_ in case of TAPBPR) and the base of the MHC I binding groove.

**Figure 7:**
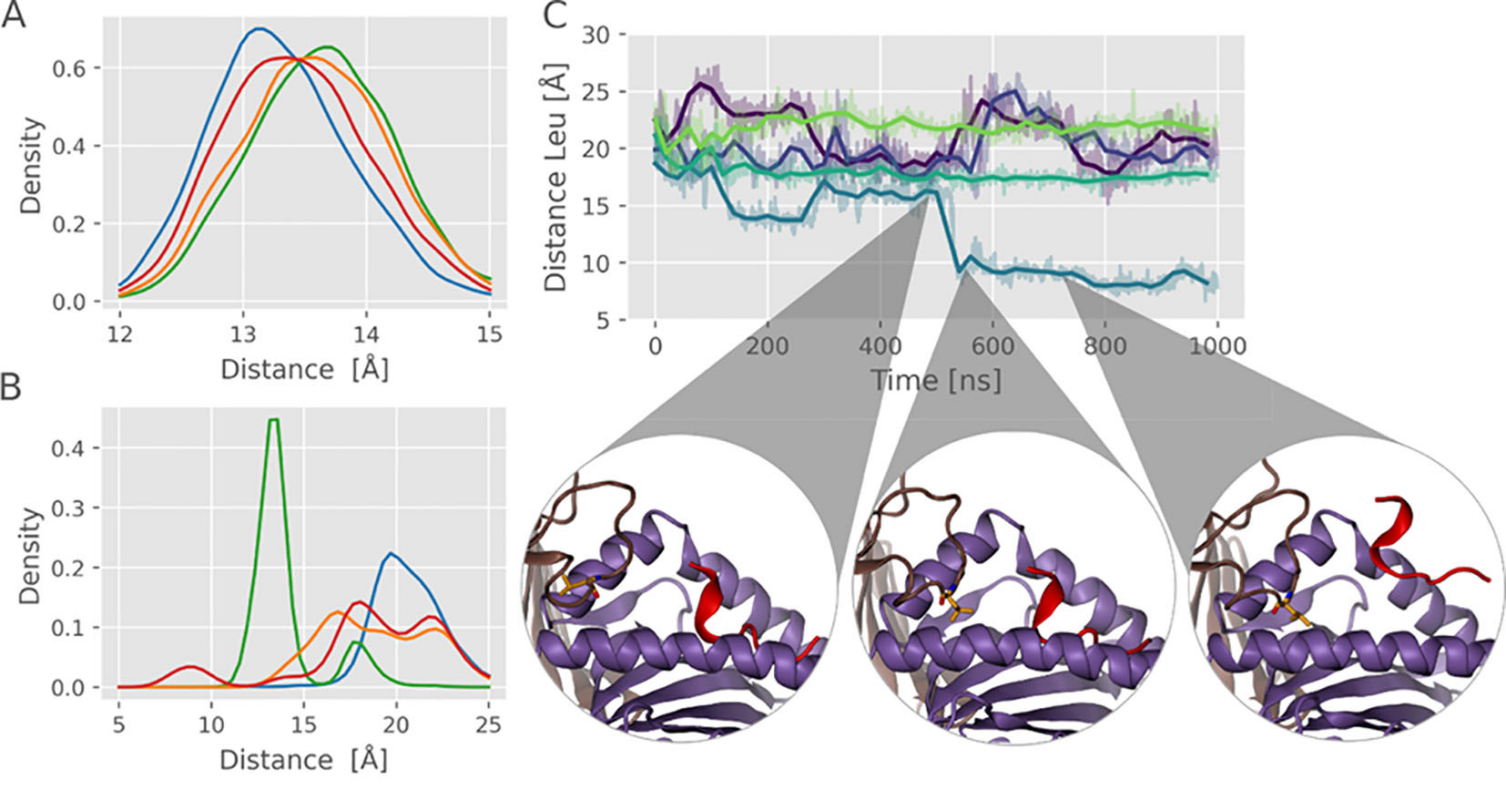
Loop dynamics and anchoring during free simulations of tapasin and TAPBPR complexes. **(A)** Distance distribution between the tapasin loop Leu_18_ residue and the F-pocket binding groove base over time, measured across five simulation replicas. Each line color represents a different peptide: green for high-affinity peptide, orange for intermediate-affinity peptide, red for low-affinity peptide, and blue for the no peptide. **(B)** same as **(A)** but for distance distribution between TAPBPR Leu_30_ and MHC F-pocket base. **(C)** Distance between TAPBPR Leu_30_ and F pocket base vs. simulation time for all 5 MD simulations with a bound low affinity peptide. Line colors correspond to replicas 1 to 5. Transparent lines represent raw data, while solid lines indicate the smoothed average (mean of 25 data points). The structural snapshots below the graph illustrate how the Leu_30_ anchors into the F-pocket binding groove as the peptide C-terminus dissociates during one of the simulations (blue line).

For tapasin, the loop segment generally shows significant conformational fluctuations, with overall slightly less fluctuations of the RMSD in the absence of peptide in most of the simulation replicas (**Supplementary Figure S6**). Apparently, the bound peptide restricts the placement of the loop segment and promotes more mobile conformations not in contact with MHC I, and increased RMSD. The recording of the distance between the central loop Leu_18_ residue of tapasin and the F pocket floor indicates that in the absence of a peptide, the Leu_18_ residue is slightly closer to the MHC pocket than when low affinity peptides are bound (**Figure 7a**). High- and intermediate-affinity peptides result in slightly greater distance between the loop leucine and the peptide pocket base. As analyzed in the previous paragraphs, in case of the low-affinity peptide, partial (C-terminal) dissociation from the F pocket was frequently observed, creating space for the loop Leu_18_ residue to move towards the F pocket (sampling similar states as seen in the absence of peptide). Hence, the result shows that a partial competition for sterical space of loop placement vs. peptide C-terminus binding is indeed possible.

However, the differences in sampled distance distributions are smaller compared to the behavior observed with TAPBPR (**Figure 7B**). Note, due to the longer loop of TAPBPR, the loop Leu_30_ residue can in principle reach the floor of the F pocket (calculated distance ~ 7.5 Å). Such a short distance and real occupation of the F pocket is sterically not possible in case of the smaller tapasin loop (except if the helices flanking the F pocket would unfold). For TAPBPR, we found in all simulation replicas also a significant mobility of the loop segment with fluctuating RMSD with respect to the start structure (**Supplementary Figure S6**). In the absence of peptide, when starting from a structure in which the loop is positioned outside the F pocket (same structure as used in case of a bound peptide), the loop Leu_30_ residue does not move into the pocket during the simulations but instead fluctuates above it, sampling various conformations (**Figure 7**). The distance histogram between Leu_30_ and floor of the F pocket reveals distances > 7.5 Å, indicating no close contact to the F pocket floor (**Figure 7b**). This agrees with NMR and MD studies of (28) that supported the placement of the loop above the F-pocket binding region. However, in simulations starting from a placement of the loop with the loop Leu_30_ residue already placed near the F pocket floor (equilibrated X-ray starting structure) it largely samples states with a close distance to the F pocket floor (**Supplementary Figure S7**). It indicates that the unrestrained MD simulations might not achieve convergence of sampling all relevant loop conformational states within the MD simulation time scale.

Nevertheless, in simulations in the presence of a low affinity peptide (starting from the same initial loop structure), the loop also samples a variety of conformations, and, interestingly, in one simulation, it adopts a conformation with the central Leu_30_ located close to the F pocket floor and the C-terminus of the low affinity peptide partially dissociated (**Figure 7C**). This observation indicates that for the TAPBPR case, loop binding can in principle replace the binding of a peptide C-terminus to the MHC I F pocket. For the bound high affinity and medium affinity peptides, the Leu_30_ residues remain outside the F pocket, sampling states with large distances to the F pocket floor (**Figure 7B**). However, movement of the Leu_30_ loop into the F pocket seems not to be the primary driving element for induced peptide dissociation, because the dissociation of the low affinity peptide C-terminus clearly occurred beforehand (**Figure 7C**). It is also important to emphasize that the results of the conformational sampling of the loop structure in TAPBPR was found to depend on the starting structure; hence, a converged sampling is not reached, and future more extensive simulations and advanced sampling techniques are required to study the role of the N-terminal loops for chaperone function.

A possible function of the loop could be to partially block the entrance to the F pocket and to prevent or interfere with rapid rebinding of an already dissociated peptide C-terminus. According to our simulation results, though, the primary driving force to destabilize the C-terminal peptide binding is the displaced α_2–1_ helix in the chaperone-bound MHC I complexes.

## Discussion

The chaperone proteins tapasin and TAPBPR are components of the peptide loading system to obtain MHC class I molecules with bound high-affinity peptide that can eventually be presented at the cell surface (1, 4, 9). For a successful immune response, it is important that only MHC class I molecules with a bound high-affinity peptide reach the surface because complexes with low-affinity peptides have a reduced life time and rapidly decay without having a chance of binding to a T cell receptor (9, 14, 45). For many but not all MHC class I alleles, the assistance of tapasin or TAPBPR for binding a high affinity peptide in the ER is essential, because these chaperones accelerate the binding process but also help to reduce binding of low affinity peptides and promote exchange with high affinity peptides (16).

In addition, tapasin (and perhaps also, TAPBPR) has a second independent function, i.e., the conformational stabilization of peptide-empty MHC I to support folding and peptide binding (46).

Recently, the experimental structures of MHC class I molecules in complex with tapasin (6, 8) and TAPBPR (21, 22) have been determined. The solved structures place an N-terminal loop element in the vicinity of the F pocket of a bound MHC class I molecule. However, at least in case of the TAPBPR complex, the quality of the electron density indicates that the loop segment might be partially disordered (21), whereas in case of tapasin, the loop is well defined and located above the F pocket (6, 8). Extensive mutagenesis and structural and biochemical studies have demonstrated the importance of the loop for the chaperone function both in case of tapasin and TAPBPR (23–26, 47). It has also been possible to identify individual loop residues necessary for allele recognition and chaperone activity (23, 24).

Another important and characteristic structural change observed in all solved MHC class I complexes with tapasin or TAPBPR is a displacement of the α_2–1_ helix towards a more open C- and F pocket binding cleft compared to a native peptide-bound MHC class I molecule. The relevance of this conformational change for the loading and editing process is still not clear. One hypothesis assumes that a more open binding region allows simply easier access of the peptides to move into the binding cleft, or it is merely a side effect of chaperone binding. A more active role to destabilize the binding of peptides by widening the F pocket regions such that it promotes dissociation of low affinity peptides has also been discussed (34, 48, 49). However, it is difficult to experimentally prove such an active role without the possibility to actively constrain the MHC class I structure to a desired conformation. Hence, the functional significance of the α_2–1_ helix opening motion in the presence of chaperones is still not clear.

We have used multiple cMD simulations and free energy simulations to systematically shift the α_2–1_ in peptide-bound and -empty MHC class I molecules and in the presence of either of the two chaperones tapasin and TAPBPR. Our simulations are based on the experimental starting structures with a mouse class I allele (H-2D^b^) in complex with human tapasin or TAPBPR. Since such complexes are functional, we expect that the principal chaperone mechanism is similar to complexes with native human class I alleles, but possible differences cannot be excluded. Also, the F pocket of the H-2D^b^ allele is relatively hydrophobic, hence, possible allele-specific differences in the mechanism cannot be excluded. Both the cMD simulations and the REUS free energy simulations revealed that a high-affinity peptide bound to the class I F pocket can pull the α_2–1_ helix towards smaller distances relative to the α_1_ helix on the opposite side of the F pocket, resulting in lower sampled helix distances compared to an empty binding cleft or in the presence of a low-affinity peptide. It indicates a competition of interactions between the peptide and the α_2–1_ helix on the one hand and an opposing interaction of the α_2–1_ helix with the chaperone on the other hand. Indeed, our calculations also revealed that the induced α_2–1_ helix movement reduces the favorable interaction of the MHC molecule with tapasin or TAPBPR and obtained an estimate in reduction of interaction energy of ~2.1-2.6 kcal/mol. Hence, binding of a high-affinity peptide to the MHC I cleft that is bound to a chaperone pulls the α_2–1_ helix slightly inwards, which reduces the binding constant to the chaperone by a factor of ~50 and might induce a release from the chaperone. Our results are in line with a proposal made over 20 years ago (49) and with recent structural studies on a TAPBPR variant with increased affinity to MHC I that achieves similar chaperone performance as the wild type although lacking the loop structure completely (34), supporting the view of a decisive role of the α_2–1_ helix movement.

However, interestingly, a final placement with the same distance as observed in the native peptide-bound MHC class I molecule was not reached even in the presence of a bound high-affinity peptide. The result also suggests that the missing final 2 Å displacement of α_2–1_ helix movement required to adopt the native MHC class I structure may occur after the MHC I detaches from tapasin or TAPBPR, and that our simulation timescales are not sufficient to capture this final movement.

Additionally, our simulations directly demonstrated that the F pocket opening by the α_2–1_ helix displacement alone is sufficient to destabilize low-affinity peptides. This was manifested by transient and frequent dissociation events of the bound low affinity peptide (and the medium-affinity peptide) especially in the C- and F pocket region in the presence of tapasin or TAPBPR. A similar effect was observed for a native MHC class I molecule including restraints to keep the F pocket arrangement close to the conformation in the presence of tapasin or TAPBPR. This proves that it does not depend on other interactions in the complex with the chaperones. In future more extensive simulation studies including several peptides, it might also be possible to observe not only dissociation but also re-association of peptides.

Tapasin and TAPBPR are known to have different preferences for MHC class I alleles and distinct preferences for supporting the loading of various types of peptides. Our results provide evidence for a general mechanism of how the α_2–1_ helix movement is coupled to peptide loading and stabilization/destabilization of peptide vs. chaperone binding. Additional interactions with the N-terminal loop located above the F pocket likely modulate the allele dependence and peptide sequence dependence not explained by our simulations. Indeed, it has been shown that sequence changes in the loop can strongly interfere with the chaperone assisted loading process and can even alter the peptide sequence preference (23, 24). In the case of MHC I bound to tapasin, our simulations indicate that the N-terminal loop stays above the F pocket and the central Leu residue does not reach the floor of the F-pocket even in the absence of a peptide. For the complex with TAPBPR, the result depends on the starting conformation. Without a bound peptide and starting from loop conformations outside the binding cleft, the central Leu residue stays outside above the F-pocket on the time scale of the MD simulations in agreement with NMR and biochemical studies (28), but a localization at the floor of the F-pocket is sterically possible as was observed in simulations starting from a placement close to the F pocket. Future, more extensive, simulation studies on the N-terminal loop structures in tapasin and TAPBPR can give new insights into the allele and peptide sequence dependence of the chaperone assisted peptide loading and editing process. In addition, possible allosteric effects due to regions not located directly at the MHC class I chaperone interface will also be investigated.

## Data Availability

Starting structures, MD trajectory files and input files are deposited in the online Zenodo repository https://doi.org/10.5281/Zenodo.17583602.

## References

[1] Blum JS Wearsch PA Cresswell P . Pathways of antigen processing. Annu Rev Immunol. (2013) 31:443–73. doi: 10.1146/annurev-immunol-032712-095910, PMID: 23298205 PMC4026165

[2] Cerundolo V Alexander J Anderson K Lamb C Cresswell P McMichael A . Presentation of viral antigen controlled by a gene in the major histocompatibility complex. Nature. (1990) 345:449–52. doi: 10.1038/345449a0, PMID: 2342577

[3] Cresswell P Bangia N Dick T Diedrich G . The nature of the MHC class I peptide loading complex. Immunol Rev. (1999) 172:21–8. doi: 10.1111/j.1600-065X.1999.tb01353.x, PMID: 10631934

[4] Springer S . Transport and quality control of MHC class I molecules in the early secretory pathway. Curr Opin Immunol. Antigen Process Cytokines. (2015) 34:83–90. doi: 10.1016/j.coi.2015.02.009, PMID: 25771183

[5] Yewdell JW . MHC class I immunopeptidome: past, present, and future. Mol Cell Proteomics. (2022) 21:1–10. doi: 10.1016/j.mcpro.2022.100230, PMID: 35395404 PMC9243166

[6] Jiang J Taylor DK Kim EJ Boyd LF Ahmad J Mage MG . Structural mechanism of tapasin-mediated MHC I peptide loading in antigen presentation. Nat Commun. (2022) 13:5470. doi: 10.1038/s41467-022-33153-8, PMID: 36115831 PMC9482634

[7] Margulies DH Taylor DK Jiang J Boyd LF Ahmad J Mage MG . Chaperones and catalysts: how antigen presentation pathways cope with biological necessity. Front Immunol. (2022) 13:859782. doi: 10.3389/fimmu.2022.859782, PMID: 35464465 PMC9022212

[8] Müller IK Winter C Thomas C Spaapen RM Trowitzsch S Tampé R . Structure of an MHC I–tapasin–ERp57 editing complex defines chaperone promiscuity. Nat Commun. (2022) 13:5383. doi: 10.1038/s41467-022-32841-9, PMID: 36104323 PMC9474470

[9] van Hateren A Elliott T . Visualising tapasin- and TAPBPR-assisted editing of major histocompatibility complex class-I immunopeptidomes. Curr Opin Immunol. (2023) 83:102340. doi: 10.1016/j.coi.2023.102340, PMID: 37245412 PMC11913765

[10] Wieczorek M Abualrous ET Sticht J Álvaro-Benito M Stolzenberg S Noé F . Major histocompatibility complex (MHC) class I and MHC class II proteins: conformational plasticity in antigen presentation. Front Immunol. (2017) 8:292. doi: 10.3389/fimmu.2017.00292, PMID: 28367149 PMC5355494

[11] Bouvier M Wiley DC . Importance of peptide amino and carboxyl termini to the stability of MHC class I molecules. Science. (1994) 265:398–402. doi: 10.1126/science.8023162, PMID: 8023162

[12] Saikia A Springer S . Peptide-MHC I complex stability measured by nanoscale differential scanning fluorimetry reveals molecular mechanism of thermal denaturation. Mol Immunol. (2021) 136:73–81. doi: 10.1016/j.molimm.2021.04.028, PMID: 34091103

[13] Madden DR . The three-dimensional structure of peptide-MHC complexes. Annu Rev Immunol. (1995) 13:587–622. doi: 10.1146/annurev.iy.13.040195.003103, PMID: 7612235

[14] Yaneva R Schneeweiss C Zacharias M Springer S . Peptide binding to MHC class I and II proteins: New avenues from new methods. Mol Immunol. (2010) 47:649–57. doi: 10.1016/j.molimm.2009.10.008, PMID: 19910050

[15] Dong G Wearsch PA Peaper DR Cresswell P Reinisch KM . Insights into MHC class I peptide loading from the structure of the tapasin-ERp57 thiol oxidoreductase heterodimer. Immunity. (2009) 30:21–32. doi: 10.1016/j.immuni.2008.10.018, PMID: 19119025 PMC2650231

[16] Praveen PVK Yaneva R Kalbacher H Springer S . Tapasin edits peptides on MHC class I molecules by accelerating peptide exchange. Eur J Immunol. (2010) 40:214–24. doi: 10.1002/eji.200939342, PMID: 20017190

[17] Solheim JC Harris MR Kindle CS Hansen TH . Prominence of beta 2-microglobulin, class I heavy chain conformation, and tapasin in the interactions of class I heavy chain with calreticulin and the transporter associated with antigen processing. J Immunol. (1997) 158:2236–41. doi: 10.4049/jimmunol.158.5.2236, PMID: 9036970

[18] Boyle LH Hermann C Boname JM Porter KM Patel PA Burr ML . Tapasin-related protein TAPBPR is an additional component of the MHC class I presentation pathway. Proc Natl Acad Sci. (2013) 110:3465–70. doi: 10.1073/pnas.1222342110, PMID: 23401559 PMC3587277

[19] Hafstrand I Aflalo A Boyle LH . Why TAPBPR? Implications of an additional player in MHC class I peptide presentation. Curr Opin Immunol. (2021) 70:90–4. doi: 10.1016/j.coi.2021.04.011, PMID: 34052734

[20] Teng MS Stephens R Pasquier LD Freeman T Lindquist JA Trowsdale J . A human TAPBP (TAPASIN)-related gene, TAPBP-R. Eur J Immunol. (2002) 32:1059–68. doi: 10.1002/1521-4141(200204)32:4<1059::AID-IMMU1059>3.0.CO;2-G, PMID: 11920573

[21] Jiang J Natarajan K Boyd LF Morozov GI Mage MG Margulies DH . Crystal structure of a TAPBPR–MHC I complex reveals the mechanism of peptide editing in antigen presentation. Science. (2017) 358:1064–8. doi: 10.1126/science.aao5154, PMID: 29025991 PMC6320693

[22] Thomas C Tampé R . Structure of the TAPBPR–MHC I complex defines the mechanism of peptide loading and editing. Science. (2017) 358:1060–4. doi: 10.1126/science.aao6001, PMID: 29025996

[23] Ilca FT Drexhage LZ Brewin G Peacock S Boyle LH . Distinct polymorphisms in HLA class I molecules govern their susceptibility to peptide editing by TAPBPR. Cell Rep. (2019) 29:1621–1632.e3. doi: 10.1016/j.celrep.2019.09.074, PMID: 31693900 PMC7057265

[24] Lan H Abualrous ET Sticht J Fernandez LMA Werk T Weise C . Exchange catalysis by tapasin exploits conserved and allele-specific features of MHC I molecules. Nat Commun. (2021) 12:4236. doi: 10.1038/s41467-021-24401-4, PMID: 34244493 PMC8271027

[25] Sagert L Hennig F Thomas C Tampé R . A loop structure allows TAPBPR to exert its dual function as MHC I chaperone and peptide editor. eLife. (2020) 9:e55326. doi: 10.7554/eLife.55326, PMID: 32167472 PMC7117912

[26] McShan AC Devlin CA Overall SA Park J Toor JS Moschidi D . Molecular determinants of chaperone interactions on MHC I for folding and antigen repertoire selection. Proc Natl Acad Sci. (2019) 116:25602–13. doi: 10.1073/pnas.1915562116, PMID: 31796585 PMC6926029

[27] McShan AC Natarajan K Kumirov VK Flores-Solis D Jiang J Badstübner M . Peptide exchange on MHC I by TAPBPR is driven by a negative allostery release cycle. Nat Chem Biol. (2018) 14:811–20. doi: 10.1038/s41589-018-0096-2, PMID: 29988068 PMC6202177

[28] McShan AC Devlin CA Morozov GI Overall SA Moschidi D Akella N . TAPBPR promotes antigen loading on MHC I molecules using a peptide trap. Nat Commun. (2021) 12:3174. doi: 10.1038/s41467-021-23225-6, PMID: 34039964 PMC8154891

[29] Sun Y Papadaki GF Devlin CA Danon JN Young MC Winters TJ . Xeno interactions between MHC I proteins and molecular chaperones enable ligand exchange on a broad repertoire of HLA allotypes. Sci Adv. (2023) 9:eade7151. doi: 10.1126/sciadv.ade7151, PMID: 36827371 PMC9956121

[30] Abualrous ET Saini SK Ramnarayan VR Ilca FT Zacharias M Springer S . The carboxy terminus of the ligand peptide determines the stability of the MHC class I molecule H-2Kb: A combined molecular dynamics and experimental study. PLoS One. (2015) 10:e0135421. doi: 10.1371/journal.pone.0135421, PMID: 26270965 PMC4535769

[31] Bailey A Dalchau N Carter R Emmott S Phillips A Werner JM . Selector function of MHC I molecules is determined by protein plasticity. Sci Rep. (2015) 5:14928. doi: 10.1038/srep14928, PMID: 26482009 PMC5224517

[32] Sieker F Straatsma TP Springer S Zacharias M . Differential tapasin dependence of MHC class I molecules correlates with conformational changes upon peptide dissociation: A molecular dynamics simulation study. Mol Immunol. (2008) 45:3714–22. doi: 10.1016/j.molimm.2008.06.009, PMID: 18639935

[33] Zacharias M Springer S . Conformational flexibility of the MHC class I α1-α2 domain in peptide bound and free states: A molecular dynamics simulation study. Biophys J. (2004) 87:2203–14. doi: 10.1529/biophysj.104.044743, PMID: 15454423 PMC1304646

[34] Sun Y Pumroy RA Mallik L Chaudhuri A Wang C Hwang D . CryoEM structure of an MHC I/TAPBPR peptide-bound intermediate reveals the mechanism of antigen proofreading. Proc Natl Acad Sci. (2025) 122:e2416992122. doi: 10.1073/pnas.2416992122, PMID: 39786927 PMC11745410

[35] Jumper J Evans R Pritzel A Green T Figurnov M Ronneberger O . Highly accurate protein structure prediction with AlphaFold. Nature. (2021) 596:583–9. doi: 10.1038/s41586-021-03819-2, PMID: 34265844 PMC8371605

[36] Reynisson B Alvarez B Paul S Peters B Nielsen M . NetMHCpan-4.1 and NetMHCIIpan-4.0: improved predictions of MHC antigen presentation by concurrent motif deconvolution and integration of MS MHC eluted ligand data. Nucleic Acids Res. (2020) 48:W449–54. doi: 10.1093/nar/gkaa379, PMID: 32406916 PMC7319546

[37] Clancy-Thompson E Devlin CA Tyler PM Servos MM Ali LR Ventre KS . Altered binding of tumor antigenic peptides to MHC class I affects CD8+ T cell–effector responses. Cancer Immunol Res. (2018) 6:1524–36. doi: 10.1158/2326-6066.CIR-18-0348, PMID: 30352798 PMC6290996

[38] Case DA Ben-Shalom IY Brozell SR Cerutti DS Cheatham TE Cruzeiro VWD III . AMBER 18. San Francisco: University of California (2018).

[39] Tian C Kasavajhala K Belfon KAA Raguette L Huang H Migues AN . ff19SB: amino-acid-specific protein backbone parameters trained against quantum mechanics energy surfaces in solution. J Chem Theory Comput. (2020) 16:528–52. doi: 10.1021/acs.jctc.9b00591, PMID: 31714766 PMC13071887

[40] Izadi S Anandakrishnan R Onufriev AV . Building water models: A different approach. J Phys Chem Lett. (2014) 5:3863–71. doi: 10.1021/jz501780a, PMID: 25400877 PMC4226301

[41] Shabane PS Izadi S Onufriev AV . General purpose water model can improve atomistic simulations of intrinsically disordered proteins. J Chem Theory Comput. (2019) 15:2620–34. doi: 10.1021/acs.jctc.8b01123, PMID: 30865832

[42] Miyamoto S Kollman PA . Settle: An analytical version of the SHAKE and RATTLE algorithm for rigid water models. J Comput Chem. (1992) 13:952–62. doi: 10.1002/jcc.540130805

[43] Hopkins CW Le Grand S Walker RC Roitberg AE . Long-time-step molecular dynamics through hydrogen mass repartitioning. J Chem Theory Comput. (2015) 11:1864–74. doi: 10.1021/ct5010406, PMID: 26574392

[44] Onufriev A Bashford D Case DA . Modification of the generalized born model suitable for macromolecules. J Phys Chem B. (2000) 104:3712–20. doi: 10.1021/jp994072s

[45] Ortmann B Copeman J Lehner PJ Sadasivan B Herberg JA Grandea AG . A critical role for tapasin in the assembly and function of multimeric MHC class I-TAP complexes. Science. (1997) 277:1306–9. doi: 10.1126/science.277.5330.1306, PMID: 9271576

[46] Garstka MA Fritzsche S Lenart I Hein Z Jankevicius G Boyle LH . Tapasin dependence of major histocompatibility complex class I molecules correlates with their conformational flexibility. FASEB J. (2011) 25:3989–98. doi: 10.1096/fj.11-190249, PMID: 21836024

[47] Hafstrand I Sayitoglu EC Apavaloaei A Josey BJ Sun R Han X . Successive crystal structure snapshots suggest the basis for MHC class I peptide loading and editing by tapasin. Proc Natl Acad Sci. (2019) 116:5055–60. doi: 10.1073/pnas.1807656116, PMID: 30808808 PMC6421438

[48] Thomas C Tampé R . MHC I assembly and peptide editing — chaperones, clients, and molecular plasticity in immunity. Curr Opin Immunol. (2021) 70:48–56. doi: 10.1016/j.coi.2021.02.004, PMID: 33689959

[49] Wright CA Kozik P Zacharias M Springer S . Tapasin and other chaperones: models of the MHC class I loading complex. Biol. Chem. (2004) 385:763–78. doi: 10.1515/BC.2004.100, PMID: 15493870

