# Supplementary material for: How conformational changes near the F pocket of MHC class I proteins mediate chaperone assisted peptide loading
Source: Front Immunol. 2025 Dec 15;16:1689803. doi: 10.3389/fimmu.2025.1689803 (PMC12745280; doi:10.3389/fimmu.2025.1689803)
Supplement: Supplementary file 1 [file DataSheet1.pdf]

Supporting Information:
How conformational changes near the F pocket of MHC class I proteins mediate chaperone assisted peptide loading

Simone Göppert[1] Sebastian Springer[2] and Martin Zacharias[1*]

[1]) Physics Department and center of Protein Assemblies, Technical University of Munich, 85748 Garching, Germany

[2]) School of Science, Constructor University, 28759 Bremen, Germany

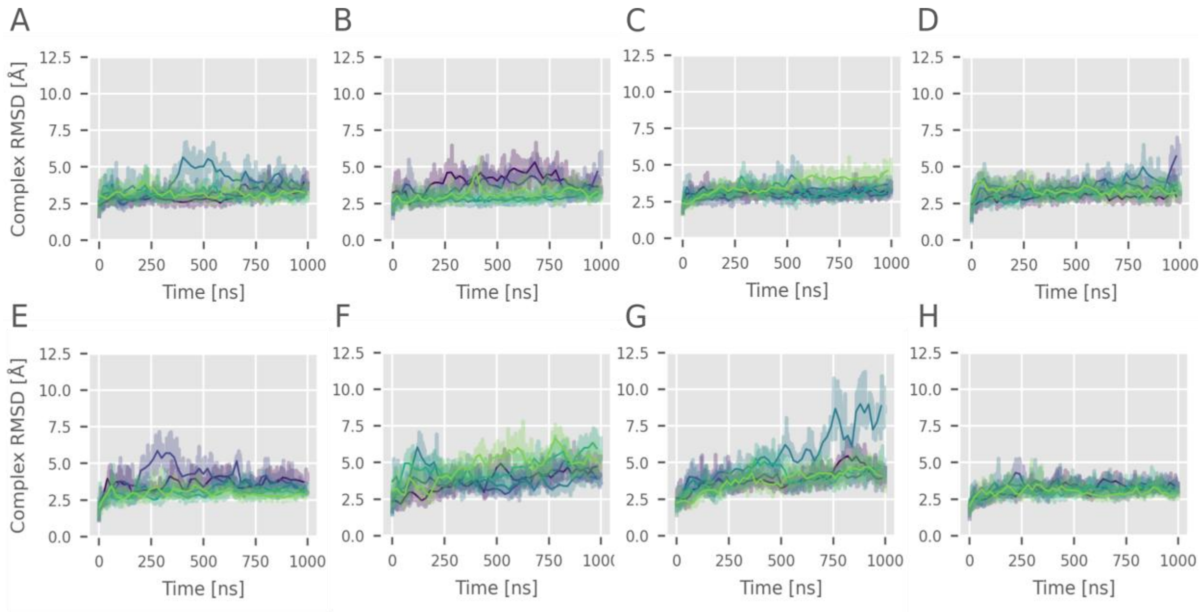

Figure S1. Backbone RMSD of the full MHC complexes vs simulation time from starting structure. In each panel results for 5 independent simulations (1000 ns, different line colors) of backbone RMSD of the entire complexes (including MHC, chaperone and peptide where applicable) are shown. (A-D) RMSD vs. time for Tapasin-bound MHC complexes, (E-H) same for TAPBPR-bound complexes. Within each row, the columns represent peptide-binding conditions: high-affinity peptide (first column: A, E), medium-affinity peptide (second column: B, F), low-affinity peptide (third column: C, G), and no peptide (fourth column: D, H). Each condition is represented by five replicas. Transparent lines represent raw RMSD values, while solid lines show the smoothed average (mean of 25 data points).

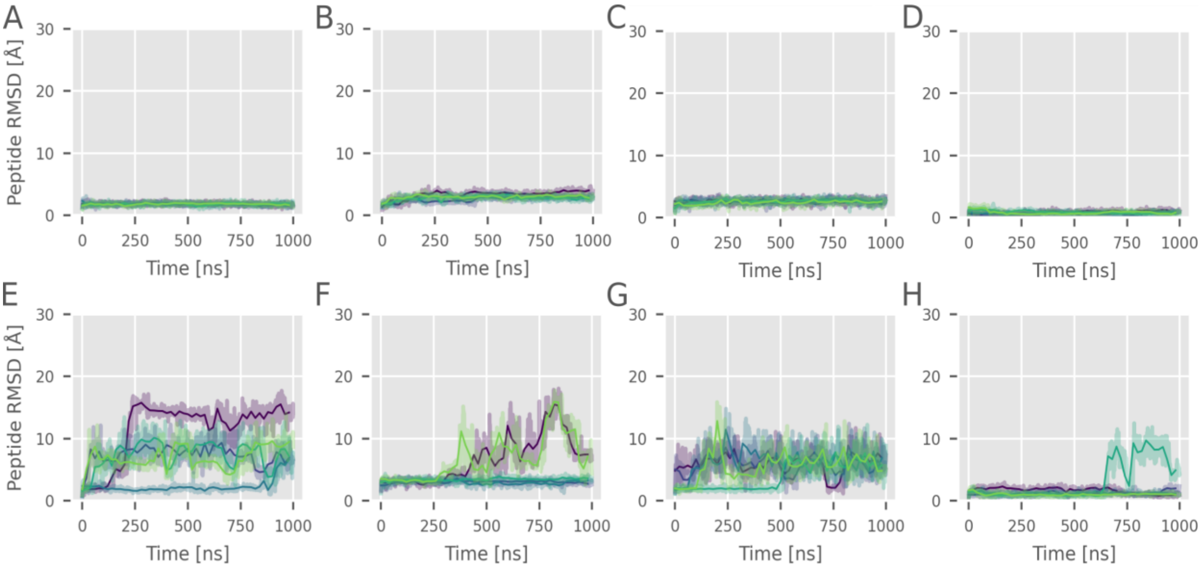

Figure S2. RMSD of the peptide C-terminus (non-hydrogen atoms of last three residues, after superposition of the MHC I peptide binding region, residues 1-175 on the starting structure) during free simulations. The plots indicate the C-terminal peptide RMSD for the high-affinity peptide in (A) MHC I complex with tapasin, (B) with TAPBPR, (C) only MHC I with open helix segments flanking the F-pocket in conformation similar to the geometries in complex with chaperones, and (D) MHC in native conformation. Panels (E–H) show the corresponding data for the medium-affinity peptide in the same order: (E) Tapasin, (F) TAPBPR, (G) MHC with open helices, and (H) locked MHC. Each colored line corresponds to one replica (replicas 1–5), with transparent lines representing raw data and solid lines showing the smoothed average (mean of 25 data points).

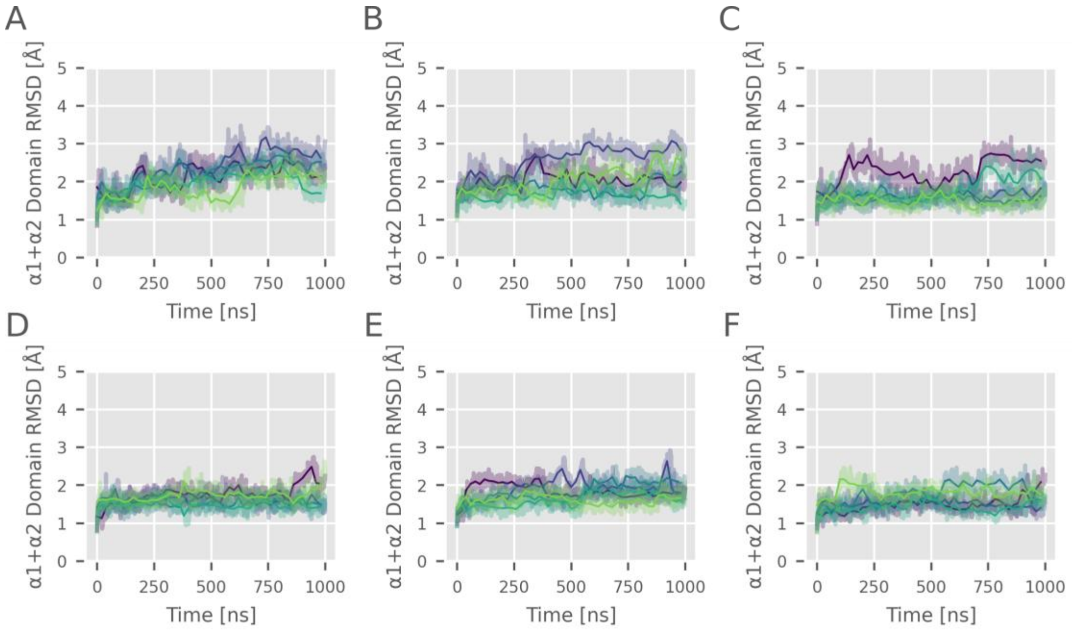

Figure S3. Backbone RMSD of the MHC α1 and α2 domain vs. simulation time. The plots show the backbone RMSD of the peptide-binding domains over 1000 ns of simulation time. The first row indicates simulations of MHC alone with restrained helix distance (A-C), and the second row shows MHC in native conformation without chaperone binding or restraints (D-F). Within each row, the columns represent peptide-binding conditions: high-affinity peptide (first column: A, D), medium-affinity peptide (second column: B, E) and low-affinity peptide (third column: C, F). Each condition is represented by five replicas (different line colors). Transparent lines represent raw RMSD values, while solid lines show the smoothed average (mean of 25 data points).

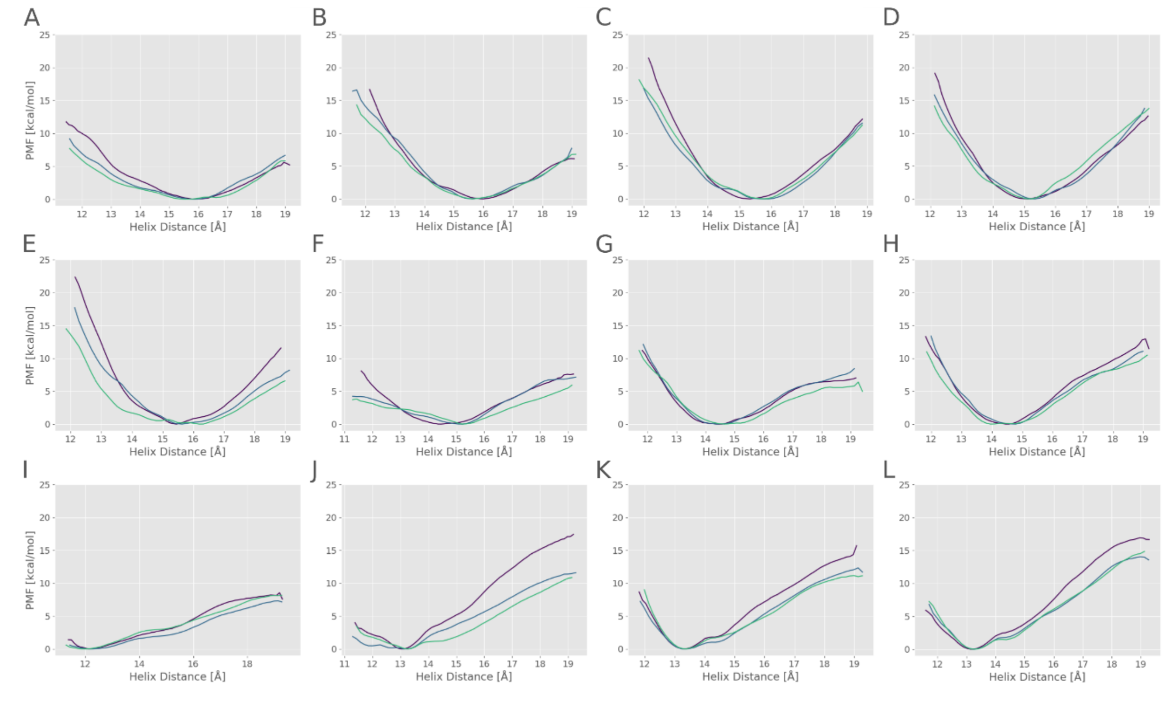

Figure S4. Convergence analysis of REUS simulations for calculating the Potential of Mean Force (PMF) along the distance between the center of mass of the Cα atoms from residue 75 to 86 for the α_1 helix segment and the Cα atoms of residues 137 to 151 for defining the $\alpha_{2\text{-}1}$ helix that flank the MHC I F-pocket. Each REUS setup consisted of 14 umbrella sampling windows covering the helix distance range, with each window simulated for 25 ns per run. The curves represent sequential REUS runs of increasing sampling time: violet for the first 25 ns, blue for the second run (extending each window to 50 ns total), and turquoise for the third run (extending each window to a total of 75 ns). (A-D) Tapasin-bound MHC complexes, (E-H) same for TAPBPR-bound complexes, (I-L). Within each row, the columns represent peptide-binding conditions: no peptide (first column: A, E, I), low-affinity peptide (second column: B, F, J), medium-affinity peptide (third column: C, G, K), and high-affinity peptide (fourth column: D, H, L).

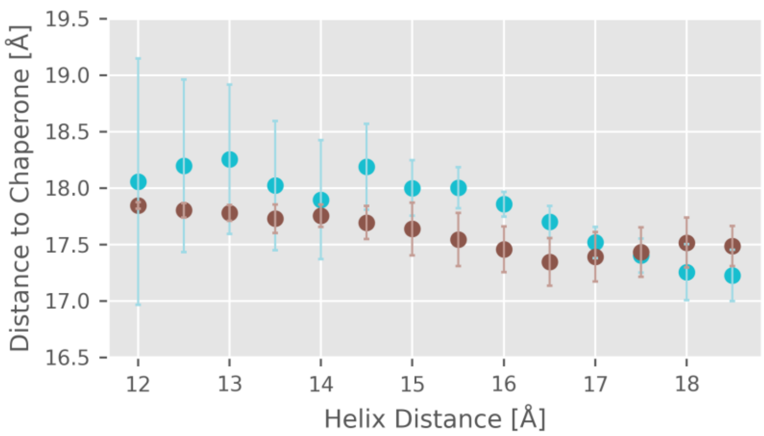

Figure S5. Average distance between the center-of-mass of the MHC class I $\alpha_{2\text{-}1}$ helix (residues: 137-151) and the center-of-masse of the $\alpha_{2\text{-}1}$ binding region of tapasin (residues 562, 575, 628, 638, cyan dots) or TAPBPR (residues 574, 586, 630, 641, brown dots) vs. target distance between α_1 helix segment and $\alpha_{2\text{-}1}$ helix during REUS MD-simulations. The selected chaperone residues are located in a continuous β-sheet region that forms a flat surface near the MHC $\alpha_{2\text{-}1}$ helix representing the main interaction interface between both proteins. The closing of the distance between α_1 helix segment and $\alpha_{2\text{-}1}$ helix induces on average a slight increase of the distance between $\alpha_{2\text{-}1}$ helix and chaperone binding region which destabilizes the association of MHC class I and chaperone but does not disrupt the interface.

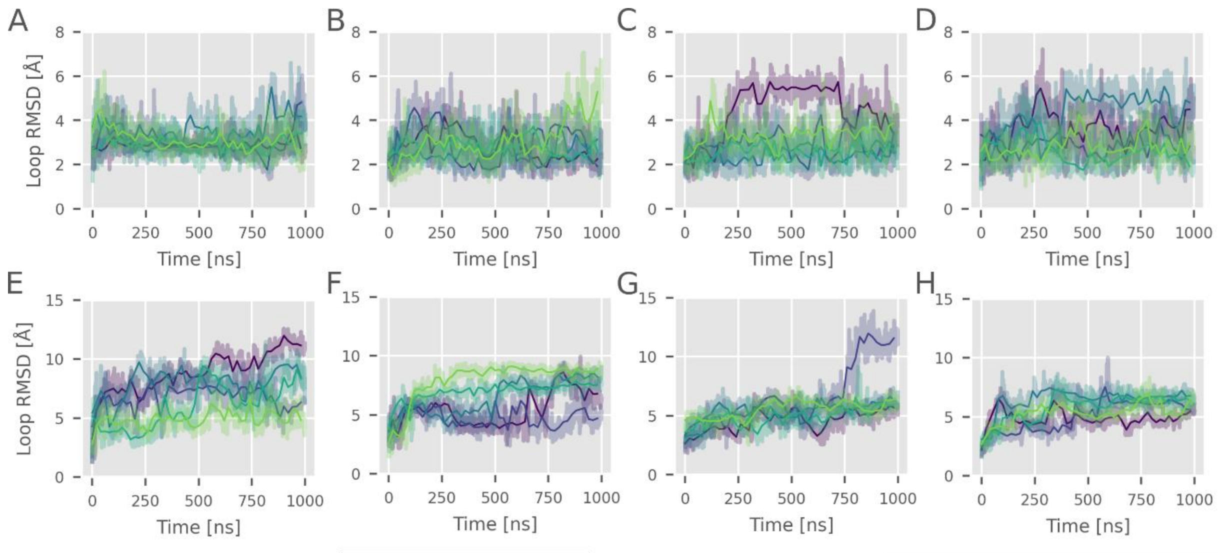

Figure S6. RMSD vs. simulation time of the Tapasin and TAPBPR loop segments. Panels show the loop RMSD over time for Tapasin with (A) no peptide, (B) low-affinity peptide, (C) medium-affinity peptide, and (D) high-affinity peptide, and for TAPBPR with (E) no peptide, (F) low-affinity peptide, (G) medium-affinity peptide, and (H) high-affinity peptide. Transparent lines represent raw values from individual replicas; solid lines show the running average (mean of 25 values per window).

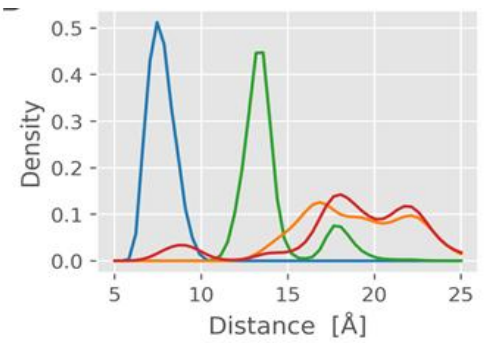

Figure S7. Distance distribution between TAPBPR Leu_{30} and MHC F-pocket base, measured across five (1µs) simulation replicas. Line colors indicate the distribution of distances between Leu_{30} and MHC F-pocket base (blue: absence of peptide, red: with bound low affinity peptide, orange: with medium affinity peptide, green: with high affinity peptide). The histograms are the same as shown in Figure 7 in the main manuscript except for the case in the absence of a peptide. In this case the simulations started from an equilibrated experimental structure (pdb5opi) with missing residues generated by Alphafold2 and the Leu_{30} already placed close to the floor of the F-pocket (distance < 10 Å). The simulation started with different initial velocities but the Leu residues remained close to the F-pocket floor (distance < 10 Å).
